# Additive Behavioral Improvement after Combined Cell Therapy and Rehabilitation Despite Long-Term Microglia Presence in Stroke Rats

**DOI:** 10.3390/ijms22041512

**Published:** 2021-02-03

**Authors:** Abdulhameed Bakreen, Miia Juntunen, Yannick Dunlop, Irene F. Ugidos, Tarja Malm, Susanna Miettinen, Jukka Jolkkonen

**Affiliations:** 1 Department of Neurology, University of Eastern Finland, FI-70210 Kuopio, Finland; abdulhameed.bakreen@uef.fi (A.B.); yannickdunlop@gmail.com (Y.D.); 2 A. I. Virtanen Institute for Molecular Sciences, University of Eastern Finland, FI-70150 Kuopio, Finland; ifernandezugidos@tulane.edu (I.F.U.); tarja.malm@uef.fi (T.M.); 3 Faculty of Medicine and Health Technology, Tampere University, P.O. Box 607, FI-33014 Tampere, Finland; miia.juntunen@tuni.fi (M.J.); susanna.miettinen@tuni.fi (S.M.); 4 Research, Development and Innovation Centre, Tampere University Hospital, P.O. Box 2000, FI-33521 Tampere, Finland

**Keywords:** stroke, microglia, inflammation, blood–brain barrier, repair, reorganization, cell therapy, rehabilitation, spontaneous recovery

## Abstract

Microglia are involved in the post-stroke immunomodulation of brain plasticity, repair, and reorganization. Here, we evaluated whether adipose-tissue-derived mesenchymal stem cells (ADMSCs) and/or rehabilitation improve behavioral recovery by modulating long-term perilesional inflammation and creating a recovery-permissive environment in a rat model of ischemic stroke. Methods: A two-way mixed lymphocyte reaction was used to assess the immunomodulatory capacity of ADMSCs in vitro. Two or 7 days after permanent middle cerebral artery occlusion (pMCAO), rats were intravenously administered ADMSCs or vehicle and housed in a standard or enriched environment (EE). Behavioral performance was assessed with a cylinder test, then we performed stereological and ImageJ/Fiji quantifications of ionized calcium-binding adaptor molecule 1 (Iba1) cells and blood–brain barrier (BBB) leakage. Results: Human ADMSCs were immunosuppressive in vitro. The cylinder test showed partial spontaneous behavioral recovery of pMCAO rats, which was further improved by combined ADMSCs and housing in EE on days 21 and 42 (*p* < 0.05). We detected an ischemia-induced increase in numbers, staining intensity, and branch length of Iba1+ microglia/macrophages as well as BBB leakage in the perilesional cortex. However, these were not different among pMCAO groups. Conclusion: Combined cell therapy and rehabilitation additively improved behavioral outcome despite long-term perilesional microglia presence in stroke rats.

## 1. Introduction

Shortly after the onset of ischemic stroke, a significant number of resident microglia are recruited into the perilesional tissue to facilitate tissue repair; however, they are also involved in mediating tissue damage [1,2]. Hours after activation, microglia change their shape from a ramified state with thin processes, via a transient hyper-ramified phenotype, toward a more stellate shape with thickened cell bodies and processes and later a large amoeboid structure with a phagocytic capacity [3]. Accompanying these morphological changes, there is a sustained increase in the proliferation and secretion of inflammatory mediators, including cytokines, chemokines, and proteolytic enzymes, collectively leading to the infiltration of monocyte-derived macrophages, neutrophils, and lymphocytes, and the proteolytic degradation of neighboring cells and the extracellular matrix as well as disturbances in the blood–brain barrier (BBB), and a worsening of neurological outcome through a feedback loop of an exacerbating inflammatory cascade [4,5,6].

Interestingly, perilesional microglia/macrophages can also assume beneficial roles, whereby they release anti-inflammatory cytokines as well as growth and repair factors that are involved in the recruitment of neural stem cells, remodeling of the extracellular matrix, tissue regeneration, as well as the scavenging and phagocytosis of cellular fragments, neurofibrillary tangles, apoptotic cells, and damaged cells [7,8,9,10,11]. Indeed, the efficient phagocytic clearance of dead cells and debris is particularly important during the regeneration and remodeling of perilesional tissue toward a restitution of function [12]. The significant contribution of microglia is also important in promoting both neuronal plasticity and subsequent behavioral recovery after stroke, as they have been shown to be involved in the proliferation and integration of endogenous neural progenitors into functional connections, modification of neuronal activity, and modulation of synaptic function [13,14]. Thus, the modulation of neuroinflammation by suppressing deleterious microglial activities while at the same time enhancing their repair and remodeling functions represents an opportunity to promote a recovery-permissive environment and improve the neurological outcome.

Cell-based therapy has emerged as a therapeutic paradigm targeting both sub-acute and chronic inflammatory processes after stroke [15,16]. Cell delivery at <24 h after stroke is aimed at providing neuroprotective immunomodulation; while delivery at ≥48 h, when the perilesional tissue is still very plastic, is aimed at promoting brain plasticity and repair mechanisms [15]. More importantly, as most cell-based studies on stroke are performed during the sub-acute phase, little is known about the inflammation response in the long-term spontaneous or cell and/or rehabilitation-induced recovery process. The in vitro anti-inflammatory and immunomodulatory effects of various cell products including adipose-tissue-derived mesenchymal stem cells (ADMSCs) have been well validated, with evidence of reduced natural killer cell proliferation and cytotoxic activity, regulation of thymus-cell proliferation, as well as modulation of inflammatory cytokines and receptor activation, possibly through direct cell–cell contact and paracrine signaling [17,18]. Long-term in vivo studies investigating the immunomodulatory effects of ADMSCs are lacking.

In this study, we hypothesized that human ADMSCs are immunomodulatory in vitro. This was tested using a two-way mixed lymphocyte reaction (MLR). Then, we speculated that the intravenous administration of human ADMSCs (48 h or 7 day) in the permanent middle cerebral artery occlusion (pMCAO) rat model of ischemic stroke, alone or when combined with rehabilitation-mimicking housing in an enriched environment (EE), would improve behavioral recovery by modulating late perilesional inflammation and creating a recovery-permissive environment. To accomplish this goal, the numbers, staining intensity, and changes in ramifications of microglia/macrophages as well as the degree of BBB leakage in the perilesional cortex were quantified by histology.

## 2. Results

### 2.1. ADMSCs Suppressed the Proliferation of Peripheral Blood Mononuclear Cells (PBMCs) In Vitro 

The proliferation of PBMCs (see Figure S1) was significantly suppressed in co-culture with ADMSCs when compared to that of PBMCs cultured without ADMSCs (*p* < 0.001).

### 2.2. Combination Therapy Improved the Long-Term Behavioral Outcome

**Figure 1:**
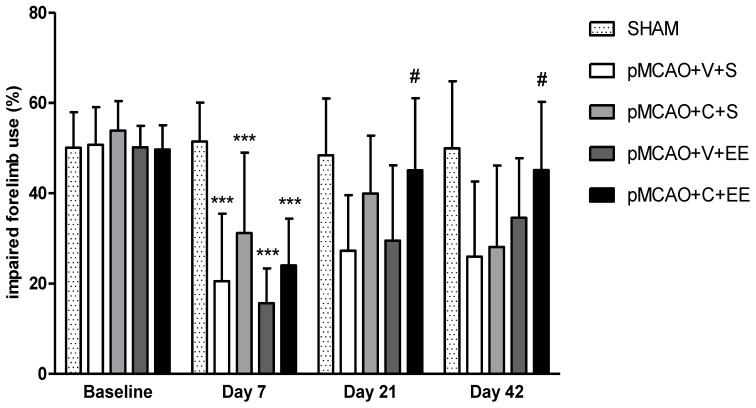
The spontaneous use of the impaired forelimb during vertical exploration was assessed with the cylinder test. There was a significant overall group effect (*p* < 0.001), time effect (*p* < 0.001), and time × group interaction (*p* < 0.001). Statistical significance at days 7, 21, and 42: *** *p* < 0.001 (compared to pooled SHAM group), # *p* < 0.05 (compared to pMCAO + V + S) (one-way analysis of variance (ANOVA) followed by Bonferroni multiple comparisons test). Data are expressed as mean ± standard deviation (SD). SHAM = sham-operated; pMCAO = permanent middle cerebral artery occlusion; V = 48 h vehicle administration; C = 48 h cell administration; S = standard housing; EE = enriched environment.

There were no differences in gross behavioral impairment assessed with Rogers’ test or in infarct size among pMCAO groups 24 h after the operation before cell infusion or housing in EE (see Figure S2). Partial spontaneous behavioral recovery in the cylinder test was observed in all rats subjected to pMCAO during the 42-day behavioral follow-up (Figure 1). There was a significant overall group effect (F (7,67) = 8.422, *p* < 0.001), time effect (*p* < 0.001) and time × group interaction (*p* < 0.001), meaning that the recovery of the use of an impaired limb in the cylinder test was different between the groups. All pMCAO groups were impaired compared to sham-operated (SHAM) animals on post-operative day 7 (*p* < 0.001). pMCAO + V + S control rats were impaired when compared to the pMCAO + C + EE combined treatment group on post-operative days 21 and 42 (*p* < 0.05).

### 2.3. Stereological Quantification Revealed a Long-Term Increase in the Number of Microglia/Macrophages in the Perilesional Cortex

**Figure 2:**
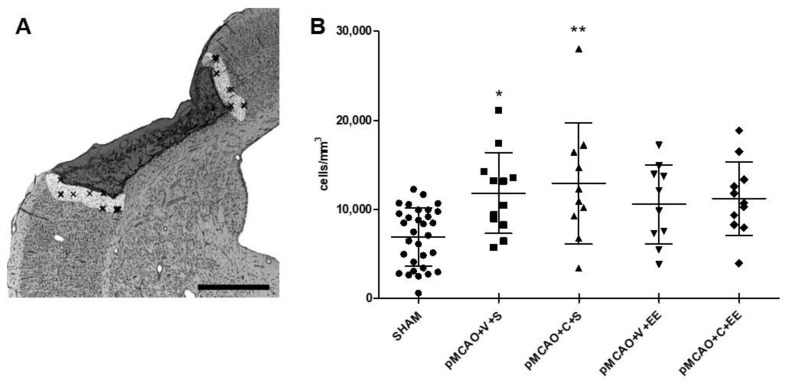
Stereological quantification of perilesional inflammation. Delineation of the perilesional cortex 200 µm from the glial scar border of the ischemic infarct core (**A**). There was an ischemia-induced increase in the number of ionized calcium-binding adaptor molecule 1 (Iba1+) cells in the perilesional cortex (**B**). Statistical significance: * *p* < 0.05, ** *p* < 0.01 (compared to the pooled SHAM group) (one-way ANOVA followed by Bonferroni multiple comparisons test). Data are expressed as mean ± SD. SHAM = sham-operated; pMCAO = permanent middle cerebral artery occlusion; V = 48 h vehicle administration; C = 48 h cell administration; S = standard housing; EE = enriched environment. Scale bar 1 mm (**A**).

Figure 2A shows how the perilesional cortex was delineated. There was a significant overall group effect (F (7,66) = 3.624, *p* < 0.01) in the density of perilesional ionized calcium-binding adaptor molecule 1 (Iba1+) cells 44 days after pMCAO (Figure 2B). The number of Iba1+ cells in pMCAO animals was increased when compared to that in SHAM animals (*p* < 0.001). There was no significant treatment effect between the vehicle-treated and ADMSC and/or rehabilitation-treated pMCAO groups (Figure 2B).

### 2.4. ADMSCs with or without Rehabilitation Did Not Change the Staining Intensity of Perilesional Microglia/Macrophages

**Figure 3:**
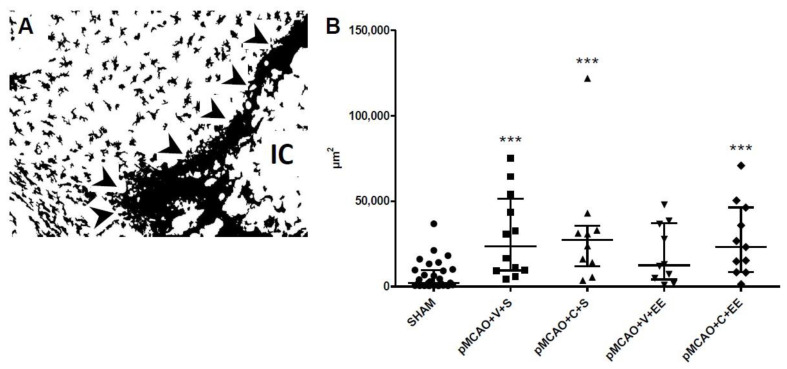
Quantification of the staining intensity of perilesional Iba1+ cells. A representative grayscale image used for quantification of ionized calcium-binding adaptor molecule 1 (Iba1) staining in a pMCAO rat (**A**). Arrows indicate glial scar. IC = ischemic core. ImageJ/Fiji quantification revealed an ischemia-induced increase in the area of Iba1 staining in the perilesional cortex (**B**). Statistical significance: *** *p* < 0.001 (compared to the pooled SHAM group) (Kruskal–Wallis H test followed by Mann–Whitney U test corrected for multiple comparisons). Data are expressed as median with interquartile range (IQR). SHAM = sham-operated; pMCAO = permanent middle cerebral artery occlusion; V = 48 h vehicle administration; C = 48 h cell administration; S = standard housing; EE = enriched environment.

There was a significant overall group effect in staining intensity of perilesional Iba1+ microglia/macrophages (*p* < 0.001). Staining intensity was increased in pMCAO + V + S, pMCAO + C + S, and pMCAO + C + EE groups compared to that in the pooled SHAM group (*p* < 0.001). There was no significant difference between the vehicle-treated and ADMSC and/or rehabilitation-treated pMCAO groups (Figure 3).

### 2.5. No Changes Were Seen in Perilesional Microglia Ramifications after ADMSCs and/or Rehabilitation

**Figure 4:**
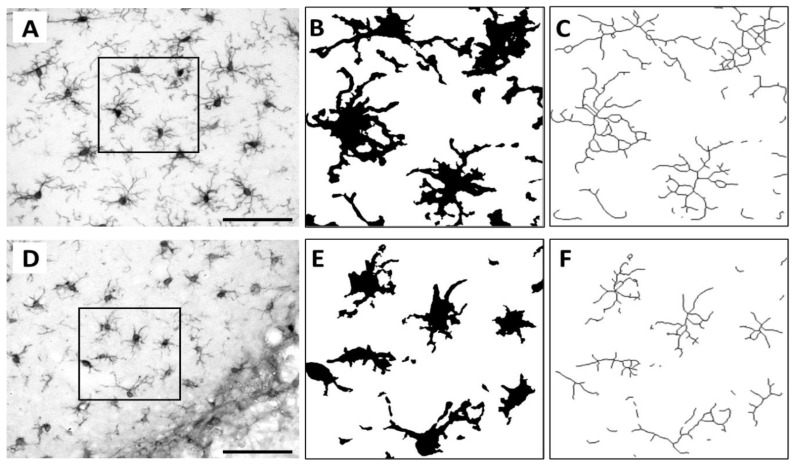
Skeletonization of perilesional Iba1+ cells. Original photomicrographs and their corresponding binary masks and skeletons showing the ramifications of the highlighted Iba1+ cells of representative SHAM (**A**–**C**) and pMCAO (**D**–**F**) animals. Scale bar 100 µm (**A**,**D**).

Skeleton analysis was carried out to quantify the morphological changes and the ramifications of microglia/macrophages in the perilesional cortex (Figure 4). There was a significant overall group effect in the average branch length of perilesional Iba1+ microglia/macrophages (*p* < 0.05). The average branch length was increased in pMCAO animals compared to that in SHAM animals (*p* < 0.001) (Table 1). However, no differences were found in the number of branches and number of branching points between SHAM and pMCAO animals. There were also no differences in all ramification parameters between vehicle-treated and ADMSC and/or rehabilitation-treated pMCAO groups (data not shown).

### 2.6. Timing of Cell Delivery Did Not Affect the Long-Term Inflammatory Response

Early (48 h) and late (7 d) cell delivery time points were compared to explore whether the timing of cell delivery exerted any effect on the long-term inflammatory response. We detected no differences in the number, staining intensity, or changes in the ramifications of perilesional microglia/macrophages on post-operative day 44 (data not shown). 

### 2.7. No Long-Term Correlations between Behavioral Outcome and Microglia Response

The spontaneous use of the impaired forelimb in the cylinder test at the end of the behavioral follow-up on post-operative day 42 did not correlate with the number (*p =* 0.458), staining intensity (*p =* 0.538), number of branches (*p =* 0.829), number of branching points (*p =* 0.692), or average branch length (*p =* 0.538) of the perilesional microglia/macrophages of pMCAO animals on post-operative day 44.

### 2.8. The Extravasation of Serum Immunoglobulin G (IgG) into Perilesional Cortex Was Increased in pMCAO Rats

**Figure 5:**
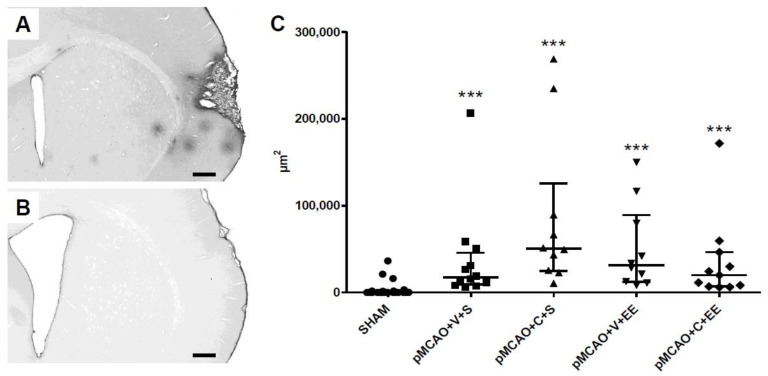
ImageJ/Fiji quantification of blood–brain barrier (BBB) leakage. Representative immunoglobulin G (IgG)-stained sections of pMCAO (**A**) and SHAM (**B**) animals. There was an ischemia-induced increase in the area of IgG staining in the perilesional cortex (**C**). Statistical significance: *** *p* < 0.001 (compared to the pooled SHAM group) (Kruskal–Wallis H test followed by Mann–Whitney U test corrected for multiple comparisons). Data are expressed as median with IQR. SHAM = sham-operated; pMCAO = permanent middle cerebral artery occlusion; V = 48 h vehicle administration; C = 48 h cell administration; S = standard housing; EE = enriched environment. Scale bar 500 µm (**A**,**B**).

The overall ipsilateral distribution of “islands” of parenchymal extravasations of serum IgG is shown for a representative pMCAO animal (Figure 5A). IgG-stained sections from SHAM animals were mostly clean (Figure 5B). There was a significant overall group effect (*p* < 0.001) in leakage of the BBB (Figure 5C). The perilesional IgG staining of pMCAO animals was increased compared to that in SHAM animals (*p* < 0.001). There was no significant difference between the vehicle-treated and ADMSC and/or rehabilitation-treated pMCAO groups.

## 3. Discussion

ADMSCs or housing in EE alone improved the impaired forelimb use of pMCAO rats in the cylinder test during a 42-day behavioral follow-up. When combined together, the treatment effect was even more pronounced. We also observed a long-lasting increase in number of Iba1-positive cells in the perilesional cortex at 44 days after stroke. However, the number of microglia, staining intensity, or changes in ramifications as well as the degree of BBB leakage in the perilesional cortex were not affected by cell treatment or rehabilitation alone or when combined together. 

### 3.1. Time Course of Microglia Behavior

Ischemic stroke induces an activation of microglia in the brain with a subsequent infiltration of peripheral macrophages [19,20]. The acute responses include altered gene and protein expression profiles and a change in the microglia phenotype toward a phagocytic function that is needed for the removal of dead tissue debris together with hematogenous macrophages, i.e., conditions that allow regenerative processes [21,22,23]. The long-term fate of microglia after stroke, however, is less well understood. Thored et al. [24] observed a transformation of ramified microglia to an amoeboid and round phenotype in the perilesional striatum; these activated myeloid cells could be detected for months. Our study revealed an increase in the number of Iba1-stained microglia/macrophages in the perilesional cortex at 44 days after pMCAO. To assess whether the partial spontaneous behavioral recovery and the treatment effect observed were associated with a change in phenotype, a semiquantitative analysis of Iba1 labeling has been conducted [25]. However, there was no difference in the staining intensity among pMCAO groups in our study. We also used skeleton analysis to quantify morphological changes and the ramifications of Iba1+ microglia and examined whether certain activational profiles were associated with the observed behavioral recovery. Interestingly, although no changes were seen in the number of branches and number of branching endpoints, the average branch length of pMCAO animals was longer when compared to that of SHAM animals. These findings may be related to the perilesional recruitment of distinct but spatially overlapping subpopulations of microglia with different phenotypes as well as in the differential activation stages involved in facilitating tissue repair [25,26]. Indeed, Ladwig et al. [25] reported Iba1+ microglia with a stellate or amoeboid morphology within sites of neovascularization after stroke; while Morrison and Filosa [26] showed that their morphological changes after stroke included both increased and decreased cell ramification, suggesting that microglia activation is a multistep, dichotomous, and stringently regulated process that results in several functional and metabolic subtypes.

Furthermore, microglia are also involved in the immunomodulation of neuroplasticity and regenerative processes after stroke, including the spontaneous rewiring of neural connections and functional responses in neurogenic niches [13]. Although the exact mechanisms are unclear, the involvement of both the pro- and anti-inflammatory activation profiles and the secretion of growth/repair factors [6,27] have been implicated in the modulation of neuronal survival and activity, synaptic remodeling, and endogenous neurogenesis [13,14]. In fact, Badimon et al. [14] recently showed that, via an adenosine-triphosphate-mediated negative feedback control mechanism, microglia modulate neuronal activity by increasing the number of their processes and extending them in a targeted manner toward the synaptic boutons of active thalamocortical projection neurons. Taken together, the emerging evidence suggests that the long-term presence of microglia/macrophages may shape the perilesional reorganization after an experimental stroke.

### 3.2. Effect of Intravenous Cell Infusion

Intravenous cell therapy is one of the most attractive strategies to enhance post-stroke recovery. Mesenchymal stem cells such as ADMSCs are safe, readily obtainable, and do not raise ethical issues [28]. Preclinical data are promising, and small early phase patient studies have shown safety and feasibility; however, the therapeutic efficacy has been modest, if present at all [29]. The translational failure strongly suggests that the therapeutic mechanisms are still poorly understood. We have previously shown that ADMSCs improve the behavioral recovery after an experimental stroke independent from angiogenesis or gliosis [30]. Here, we hypothesized that the strong immunosuppressive properties of ADMSCs might be beneficial in limiting the long-term inflammatory reaction that accompanies an ischemic injury. Indeed, our in vitro data support this concept, but this was not reflected in the number, staining intensity, or morphological changes of Iba1+ cells in ADMSC-treated pMCAO rats. Delivery time (2 vs. 7 days) did not explain the results.

Previous studies have produced somewhat conflicting results. Both human and rat bone-marrow-derived mesenchymal stem cells (BMMSCs) transplanted via the tail vein improved motor behavior recovery of pMCAO rats, accompanied by enhanced microglia activation [31]. Furthermore, intravenous allogenic BMMSCs improved the behavioral recovery and increased Iba1+ and cluster of differentiation 68 (CD68+) cells after pMCAO [32]. In contrast, BMMSCs administered 7 days after pMCAO in old rats improved the long-term neurological outcome but decreased the numbers of Iba1+ cells [33]. More interestingly, the decrease persisted for at least 4 months, again suggesting some involvement in the late recovery. Furthermore, the improved functional recovery and a decrease in the numbers of perilesional Iba1+ cells were reported 4 weeks after the intra-arterial delivery of human ADMSCs into transient MCAO rats [34]. ADMSCs were also shown to decrease perilesional Iba1 staining in hyperglycemic stroke rats [35]. These controversial results of transplanted cells on microglia number and activation might be partly due to differences in the route, timing and dose of transplantation, follow-up time, stroke model, as well as staining and counting techniques. In this study, however, ADMSCs did not have a significant effect on the number or phenotype of microglia/macrophages in the perilesional cortex.

### 3.3. Effect of Rehabilitation

Rehabilitative therapies after stroke promote functional recovery although very little is known about the underlying cellular mechanisms. In particular, the effects of rehabilitative training on microglia, which play an important role in the pathophysiology of cerebral ischemia, are rather poorly understood. Housing in an EE, providing social, visual, and sensorimotor stimulations, including a voluntary running wheel exercise, is commonly used as a non-specific model for rehabilitation [36]. Keiner et al. [37] showed that an EE and reaching training both strongly reduced the proliferation of microglia/macrophages in the perilesional zone. A reduced microglial response was associated with an improved functional outcome: two toll-like receptors (TLRs), TLR2 and TLR4, are thought to play important roles [38,39]. In a controlled cortical impact mouse model of traumatic brain injury, voluntary exercise initiated on week 4, but not on week 1, resulted in a decrease in the number of hypertrophied microglia with large cell bodies as well as shorter and thicker processes and an associated decrease in lesion size; there was also an increase in plasticity markers and an increase in neurogenesis and an improvement in the cognitive recovery 3 months post-trauma [40]. In the present study, we found that long-term behavioral recovery of pMCAO rats after housing in an EE was not associated with the number, staining intensity, number of branches, number of branching points, or average branch length of perilesional Iba1+ microglia/macrophages. This discrepancy reinforces the fact that more studies are needed to understand the effects of different rehabilitation paradigms on microglia subtypes and activation profiles at different time points.

### 3.4. Combined Cell Therapy and Enriched Environment

Combination therapies to promote stroke recovery are an unexploited strategy that can maximize the treatment effects. Multiple therapeutic targets can be simultaneously activated with extended time windows. The combination can yield a greater effect than the sum of their individual effects (synergistic effect) or the effects of individual therapies do not influence one another (additive effect), with the result being equivalent to the sum of effects of each therapy separately [41]. It is also claimed that cell therapy may open plasticity mechanisms and prime the brain to follow a rehabilitation-induced stabilization of newly formed functional connections. Thus, the sequential order of therapies is crucial as suggested by Wahl and Schwab [42]. In the case of an EE as a rehabilitation model, the modality of the paradigm is also crucial. Indeed, it has been shown that a three-phase intervention strategy, wherein the EE cage was periodically rearranged according to three post-stroke phases, promoted long-term neurogenesis, angiogenesis, neurovascular remodeling, spatial learning, and memory in pMCAO rats [43].

To our knowledge, there are no studies on how combined cell therapy and rehabilitation affect microglia. Sasaki et al. [44] showed that combination therapy exerted a synergistic effect, significantly reducing the infarct volume, increasing corpus callosum thickness, and inducing synaptogenesis, thereby increasing the recovery of motor functions. On the other hand, housing photothrombotic stroke mice in an EE during the chronic phase promoted functional recovery but without synergistic effects with a sub-acute ephrin type-A receptor 4–targeted therapy [45]. Liebigt et al. [46] combined rehabilitative training and indomethacin or minocycline treatment and showed strongly improved sensorimotor performance and a significantly reduced number of proliferating microglia as compared to reaching training alone. The improved long-term behavioral recovery of pMCAO rats after combined ADMSCs and housing in an EE was not associated with the number, staining intensity, or change in the skeleton morphology of perilesional microglia/macrophages, which were examined in this study. These outcomes emphasize the complex nature of study design and the challenges faced when attempting to distinguish combinatorial effects, e.g., due to the limited statistical power. Therefore, a routine investigation of cell therapy in combination with neurorehabilitation was recommended by the Stem Cells as an Emerging Paradigm in Stroke 4 (STEPS 4) committee only when significant additional therapeutic effects would be expected from the combination or when the combination played a crucial role in the mode of action [47].

### 3.5. Long-Term Perilesional BBB Leakage in pMCAO Rats

Early after stroke, parenchymal extravasation of immune cells and serum proteins into perilesional tissue is increased due to vascular permeability and BBB dysfunction [48,49]. There is also evidence of a less pronounced but persistent and long-lasting BBB permeability, which has been implicated in excessive edema formation, hemorrhagic transformation, formation of microthrombi, and chronic inflammation, highlighting the role of an incomplete BBB recovery in the progression of ischemic injury [48,50,51,52]. In addition, the newly formed blood vessels needed for perilesional remodeling after stroke are immature, unstable, and leaky [48] and can enhance and prolong the inflammatory response. The present results from IgG staining were in line with BBB damage after stroke and might contribute to the infiltration of peripheral immune cells into brain parenchyma, explaining at least partly, the long-term presence of microglia in the perilesional cortex. ADMSCs alone or together with rehabilitation did not promote vascular stabilization or BBB recovery as previously suggested [53]. 

### 3.6. Limitations of the Study

Histological analysis of microglia morphology is a classical approach to distinguish different phenotypes. Microglia ramifications to detect complex and subtle changes in morphology and activation in response to ischemic injury can be quantified by Sholl or skeleton analysis. A notable limitation of our skeleton analysis was the variability in staining intensity, which probably affected the quantification of the ramifications of the microglia processes along their entirety [54,55]. In addition, a whole photomicrographic skeleton analysis is not optimal in conditions where there are microglia/macrophages with different morphologies within the same region of interest [55]. Histological staining at the late time point was not sensitive to detect microglia polarization (e.g., triggering receptor expressed on myeloid cells 2, arginase). Thus, morphologic characterization should be complemented by fluorescence-activated cell sorting analysis and single-cell ribonucleic acid sequencing to detect distinct subclasses of microglia. 

## 4. Materials and Methods 

### 4.1. Animals

Adult male Sprague Dawley rats from Envigo (Horst, The Netherlands) (*n* = 97, operation weight 274–340 g) were maintained throughout the experiment in a controlled environment (temperature 20 ± 1 °C; humidity 50–60%; 12-h light period 07:00–19:00) with access to food and tap water ad libitum. This project was approved by the Animal Ethics Committee (the license numbers ESAVI/7648/04.10.07/2014 and ESAVI/9539/04.10.07/2017, Hämeenlinna, Finland) and the animal care procedures were conducted according to the guidelines set by the European Community Council Directives 86/609/EEC. All efforts were made to minimize pain and suffering of animals.

### 4.2. Isolation, Culturing, and Immunophenotyping of Human ADMSCs 

The adipose-tissue-derived stem cells (Master Cell Bank/Stock no.1—Donor RESSTORE01, Batch no.: 591133643763) were isolated as previously described [56] and cultured as previously described [30]. ADMSCs showed a mesenchymal stem cell–like immunophenotype as previously described [30]. Cells at passages (PX) +2 were used for two-way MLR studies while cells at PX +2–3 were used for the in vivo experiments.

### 4.3. Assessment of Immunomodulatory Potential of ADMSCs In Vitro 

For two-way MLR studies, allogeneic human PBMCs were isolated from buffy coat samples from two donors with density gradient centrifugation using Ficoll-Paque PLUS (density 1.077 g/mL, GE Healthcare, Little Chalfont, Buckinghamshire, UK) according to the manufacturer’s instructions. PBMCs were cryopreserved until co-cultures in two-way MLRs. Two-way MLRs were performed as previously described by McIntosh and colleagues [57]. Briefly, ADMSCs were co-cultured with PBMCs for 4 days, and PBMC proliferation was measured with bromodeoxyuridine-enzyme-linked immunosorbent assay (BrdU-ELISA) analysis. Prior to the two-way MLR experiments, ADMSCs were licensed for 48 h with 10 ng/mL interferon-gamma (R&D Systems). For two-way MLR, PBMCs from two donors were thawed, counted, and mixed together in equal amounts to activate the proliferative response of each PBMC line. When examining the effect of ADMSCs, 120,000 ADMSCs were plated into 24-well plates (Nunc, Thermo Scientific, Roskilde, Denmark) and MLR was added into ADMSC cultures (1.2 million PBMCs from two donors in total/well). For controls, MLRs were cultured without ADMSCs and ADMSCs were cultured alone. Quadruplicate reactions were performed for each group. After 3 days of culture, 10 mM BrdU was added to mono- and co-cultures, and cells were cultured for an additional 16 h. The PBMC proliferation rate was measured by BrdU analysis, which was performed on day 4 using BrdU-ELISA (Roche Applied Science, Penzberg, Germany) according to the manufacturer’s instructions. The absorbances of three pipetting triplicates from each well were measured using a microplate reader (Victor 1429 Multilabel Counter; Wallac, Turku, Finland). Prior to statistical analysis, the following calculations were performed for the raw data of BrdU-ELISA; the average absorbance values were calculated for each group, the average absorbance of ADMSCs alone was subtracted from the co-culture of ADMSC and MLR average values for each well and thereafter, the co-culture values were divided by the average MLR value without the ADMSCs, considered as the baseline response in the two-way MLR. According to the performed calculations, in two-way MLRs, value 1 represents a baseline response and reaction values <1 indicate PBMC suppression. These calculations were modified from the procedure described by McIntosh [57].

### 4.4. In Vivo Study Design

**Figure 6:**
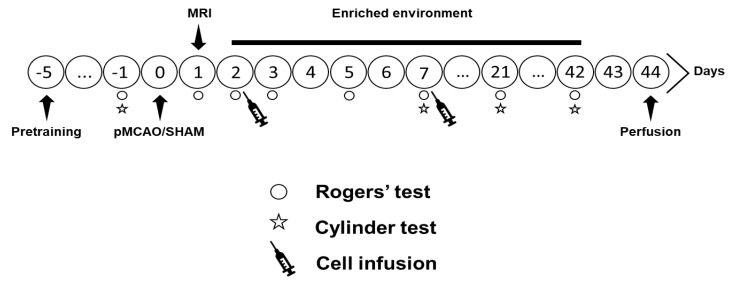
Study design.

The study design is shown in Figure 6. Focal cerebral ischemia was induced by the pMCAO model as previously described [30]. Shortly, rats were anesthetized with isoflurane and a 2–3 mm hole was drilled on the skull above the middle cerebral artery (MCA), which was electrocoagulated. Subsequently, both common carotid arteries (CCAs) were occluded with micro-aneurysm clips for 60 min. SHAM rats underwent all procedures except the occlusions of the MCA and CCAs. Magnetic resonance imaging (MRI) was performed 24 h after operation to check the size and location of the infarct (see Figure S2). Animals with infarct size <20 mm^3^ (*n* = 5) and >150 mm^3^ (*n* = 2) were excluded from data analysis. Rogers’ test, done to observe gross sensorimotor function, also showed equal behavioral impairment between groups at 24 h post-stroke (see Figure S2). Two or 7 days after pMCAO, isoflurane-anesthetized rats were slowly infused with two million ADMSCs in 1 mL 0.9% saline through the tail vein, while vehicle groups were given 1 mL 0.9% saline. After cell infusion, half of the animals were moved to a rehabilitation-mimicking EE. Each EE consisted of two large metal cages (61 × 46 × 46 cm) connected by a larger tunnel and included ladders, smaller tunnels, shelves, a running wheel, as well as novel objects that were changed twice a week. The EE provided non-specific sensorimotor, social, and visual stimulations. The control animals were housed in groups of three rats per standard cage (53 × 32.5 × 20 cm). The experimental groups were: SHAM + V + S (*n* = 8), SHAM + C + S (*n* = 8), SHAM + V + EE (*n* = 8), SHAM + C + EE (*n* = 8), pMCAO + V + S (*n* = 12), pMCAO + C + S (*n* = 10), pMCAO + V + EE (*n* = 10), pMCAO + C + EE (*n* = 11), pMCAO + V7 + EE (*n* = 8), and pMCAO + C7 + EE (*n* = 7), where V = 48 h vehicle delivery, C = 48 h cell delivery, V7 = 7 day vehicle delivery, C7 = 7 day cell delivery, S = standard housing, EE = enriched environment. SHAM groups were pooled together for statistical analysis.

### 4.5. Behavioral Testing

The cylinder test was used to measure spontaneous forelimb use and the imbalance between the non-impaired and impaired forelimbs in pMCAO rats [30]. It shows some spontaneous recovery, while being at the same time sensitive to detect long-term impairment and treatment responses [58]. Exploratory activity in the cylinder was video recorded for 3 min and later analyzed using a program with slow-motion capabilities. The number of contacts on the cylinder by either the impaired or the non-impaired forelimb or both forelimbs was counted. The imbalance in forelimb use was calculated as: ((use of impaired forelimb + 0.5 × use of both forelimbs) ÷ (total contacts)) × 100%.

### 4.6. Histology

The animals were perfused on post-operative day 44 with 0.9% saline followed by 4% paraformaldehyde (PFA) in 0.1 M phosphate buffer (PB), pH 7.4. The brains were then carefully removed from the skull, post-fixed with 4% PFA, and cryoprotected with 30% sucrose. A sliding microtome was used to cut 35-µm-thick coronal sections, which were then stored in antifreeze solution at −20 °C. Sections were stained for Iba1+ microglia/macrophages. Systematically sampled sections (a random start and covering the entire infarct) were selected for stereological analysis of the number of microglia/macrophages in the perilesional cortex; while three adjacent sections in the middle of the lesion were selected for ImageJ/Fiji quantification of Iba1 staining intensity, skeleton analysis of the change in microglia/macrophage morphology, and analysis of IgG staining intensity for BBB leakage. 

#### 4.6.1. Iba1 Immunohistochemistry 

Free-floating sections were first washed in 0.1 M PB (3 × 15 min) at room temperature (RT) and then overnight with gentle agitation in a cold room. On the next day, endogenous peroxidase activity was blocked with 0.3% hydrogen peroxide (H_2_O_2_); non-specific binding blocked with 10% normal goat serum (NGS) in Tris buffered saline + Triton (TBS-T) for 30 min, and sections were incubated on a shaker table at RT with the primary antibody mouse anti-Iba1 (Millipore) at 1:1000 in 5% NGS-TBS-T for about 21 h 30 min, with the secondary antibody biotinylated anti-mouse immunoglobulin G (IgG) (Vector) made in goat at 1:500 in TBS-T for 2 h and streptavidin–horseradish peroxidase (S-HRP) conjugate (GE Healthcare, Little Chalfont, Buckinghamshire, UK) at 1:1000 in TBS-T for 2 h. Sections were rinsed in TBS-T (3 × 5 min) before and after all blocking and antibody incubation steps. Afterward, the sections were carefully developed with filtered nickel-intensified 3,3′-diaminobenzidine (DAB) (Sigma, St. Louis, MI, USA) for approximately 3 min with any excess DAB rinsed off with PB (3 × 4 min). The specimens were left in the cold room overnight; mounted the next day, and dried overnight in 37 °C. Finally, they were cleared in xylene (2 × 5 min) and coverslips were mounted using Depex.

#### 4.6.2. IgG Immunohistochemistry 

Free-floating sections were washed in 0.1 M PB for (3 × 5 min); rinsed in 1% H_2_O_2_ for 15 min at RT to quench endogenous peroxidase activity; blocked with 2% NGS-TBS-T for 2 h at RT; treated with biotinylated sheep anti-rat IgG (AbD Serotec, Oxford, UK) at 1:200 in TBS-T for 48 h at 4 °C on a shaker table (sections without antibody incubation were used as negative controls); incubated in S-HRP conjugate (GE Healthcare, Little Chalfont, Buckinghamshire, UK) at 1:1000 in TBS-T for 1 h; and then developed with filtered nickel-intensified DAB (Sigma, St. Louis, MI, USA) for 4 min. Sections were rinsed in TBS-T (3 × 5 min) before and after all blocking and antibody incubation steps. Excess DAB was rinsed away with 0.1 M PB (3 × 4 min) before the tissues were mounted and then kept in 37 °C to dry overnight. The mounted sections were cleared in xylene (2 × 5 min) and finally, Depex was used to mount cover glasses.

### 4.7. Histological Analyses

Stereological analysis was done using a Stereo Investigator software (MicroBrightField, Inc., Williston, VT, USA) attached to an ECLIPSE E600 microscope (Nikon, Tokyo, Japan) via a 3-Chip charged-coupled device color video camera (QImaging, Surrey, BC, Canada). A motorized stage with an attached microcator (Heidenhain EXE 610C), providing a 0.1 μm resolution in the Z axis, was mounted on the microscope. The optical fractionator technique [59] was used to measure the total number of Iba1-labeled cells in the perilesional area. The perilesional area was defined as a 200-μm-wide cortical zone directly surrounding the glial scar, which was defined as an aggregation of glial cells. The parameters used included a tissue cut thickness of 35 μm, an average mounted thickness of 20 μm, a counting frame of 100 μm × 100 μm, a counting/sampling grid of 250 μm × 250 μm, and a 20-μm-high dissector cube. A 4× objective (numerical aperture 0.10) was used to trace the perilesional area, while a 40× objective (numerical aperture 0.75) was used to count the number of Iba1-positive cells. An Iba1+ cell was counted when the cell soma did not intersect with the uppermost focal plane (exclusion plane) as well as the lateral exclusion boundaries of the counting frame. The perilesional reference volume was determined by multiplying the sum of the traced perilesional areas for each section with the distance between sampled sections. The total number of Iba1+ cells was calculated using the following equation: N_total_ = ΣQ × 2 × 1/ssf × 1/asf × 1/tsf, where ΣQ is the total count per animal; ssf is the section sampling fraction; asf is the area sampling fraction (ratio of the area of the counting frame to the area of the sampling grid); tsf is the tissue sampling fraction. The number of Iba1-labeled cells was then related to 1 mm^3^ of perilesional volume.

Image analyses were done with the open-source ImageJ/Fiji software: ImageJ 2.0.0/1.53c/Java 1.8.0_172 (64-bit). Photomicrographs of sections were created with the Hamamatsu digital slide scanner (model: C12000-02, source lens 40×). Prior to analysis, the infarcted regions of three Iba1-stained and IgG-stained sections per animal were cropped with the Hamamatsu viewer software (NDP.view2) at magnifications 3× or 20× for staining intensity and skeleton analyses, respectively. After converting the images from red–green–blue color to 8-bit grayscale, thresholds were manually adjusted to generate images with a maximum of Iba1+ cells and a minimum of background artefacts. In the Iba1 staining intensity analysis, the outside border of the glial scar was outlined as precisely as possible and a specifically written macro was run to outline a 200-µm-wide perilesional cortex away from the glial scar border. The area (µm^2^) of only the thresholded pixels was then measured to derive the overall morphology of Iba1+ cells in the perilesional cortex. IgG staining intensity was quantified in a similar way. 

In the skeleton analysis, measurements were done in a 140 μm × 140 μm box placed on the glial scar border to ensure that the perilesional cortex did not exceed 200 μm. The binary mask generated was skeletonized and analyzed to derive the number of branches, number of branching points, and average branch length. The sum of all the values of each measured parameter was then divided by the number of cells in the 140 μm × 140 μm box to estimate the average ramification parameter per 1 perilesional Iba1+ cell [60].

### 4.8. Statistical Analysis

The independent-samples T test in IBM SPSS Statistics 27 for Windows was used to analyze the statistical difference in MLR absorbance values between groups. Repeated measures ANOVA was used to analyze the cylinder test data. One-way ANOVA followed by Bonferroni test was utilized to analyze the statistical differences between groups at days 7, 21, and 42 and the group differences in the number of Iba1+ cells. Kruskal–Wallis H test, followed by Mann–Whitney U test corrected for multiple comparisons, was used to analyze the staining intensity and skeleton analysis of Iba1+ cells as well as the IgG staining intensity for BBB leakage. Pearson and Spearman correlations were used to examine the relationships between behavioral impairment and the number, staining intensity, number of branches, number of branching points, or average branch length of Iba1+ cells. Data are expressed as mean ± SD or median with IQR.

## 5. Conclusions

Combining cell-based therapies with conventional rehabilitation represents an attractive strategy to enhance stroke recovery and to maximize the treatment effects. Indeed, emerging evidence suggests that cell therapies can create a highly plastic, regenerative state in which newly formed functional neural connections can be spatially directed and stabilized by subsequent rehabilitation [41]. The mechanisms promoting or inhibiting brain repair after stroke should be better understood to take the promising innovative combination treatments to clinical testing. The long-term inflammatory response suggests that the immune system is a critical player in the restoration and maintenance of brain function after an ischemic injury. However, microglia were not affected by ADMSCs and/or rehabilitation in this study. This does not exclude the subtle changes in microglia phenotypes, activation states, as well as functional and metabolic subtypes, which should be studied in future. In addition, a parallel spontaneous behavioral recovery and a long-term increase in microglia numbers after stroke suggest that microglia may provide structural support or shape the perilesional landscape and reorganization together with new blood vessels and glial cells [30]. Understanding the modulation of these processes might offer new therapeutic strategies with a longer window of opportunity to improve functional recovery after stroke.

## Figures and Tables

**Table 1:** Quantification of microglia branch length (µm).

SHAM	2.94 (2.63; 3.26)
pMCAO + V + S	3.33 (2.88; 4.35)
pMCAO + C + S	3.69 (3.42; 4.25) **
pMCAO + V + EE	3.31 (3.07; 3.92)
pMCAO + C + EE	3.32 (3.07; 3.57)

Data are presented as median with IQR. Statistical significance: ** *p* < 0.01 (compared to the pooled SHAM group) (Kruskal–Wallis H test followed by Mann–Whitney U test corrected for multiple comparisons). SHAM = sham-operated; pMCAO = permanent middle cerebral artery occlusion; V = 48 h vehicle administration; C = 48 h cell administration; S = standard housing; EE = enriched environment.

## Data Availability

The data presented in this study are available from the corresponding author.

## Supplementary Materials

Supplementary Materials can be found at https://www.mdpi.com/1422-0067/22/4/1512/s1. Figure S1. Two-way mixed lymphocyte reaction (MLR): The proliferation of peripheral blood mononuclear cells (PBMCs) was used to assess the immunomodulatory capacity of adipose-tissue-derived mesenchymal stem cells (ADMSCs) in vitro. The average absorbance value of PBMC proliferation in ADMSC + PBMC co-culture was significantly lower than that of PBMC-only monoculture (three pipetting triplicates from quadruplicate reactions per group). Statistical significance: *** *p* < 0.001. ADMSCs = adipose tissue-derived mesenchymal stem cells; PBMCs = peripheral blood mononuclear cells. Figure S2. Infarct size 24 h after pMCAO. There was no difference among experimental groups. Gross behavioral impairment assessed with Rogers’ test was not different among pMCAO groups.

## Abbreviations

ADMSCs	Adipose tissue-derived mesenchymal stem cells
ANOVA	Analysis of variance
BBB	Blood–brain barrier
BMMSCs	Bone-marrow-derived mesenchymal stem cells
BrdU	Bromodeoxyuridine
CCA	Common carotid artery
CD68	Cluster of differentiation 68
DAB	3,3′-Diaminobenzidine
EE	Enriched environment
ELISA	Enzyme-linked immunosorbent assay
H_2_O_2_	Hydrogen peroxide
Iba1	Ionized calcium-binding adaptor molecule 1
IgG	Immunoglobulin G
IQR	Interquartile range
MCA	Middle cerebral artery
MLR	Mixed lymphocyte reaction
MRI	Magnetic resonance imaging
NGS	Normal goat serum
PB	Phosphate buffer
PBMC	Peripheral blood mononuclear cell
PFA	Paraformaldehyde
pMCAO	Permanent middle cerebral artery occlusion
PX	Passages
RESSTORE	Regenerative Stem Cell Therapy for Stroke in Europe
RT	Room temperature
SD	Standard deviation
SHAM	Sham-operated
STEPS 4	Stem Cells as an Emerging Paradigm in Stroke 4
S-HRP	Streptavidin–horseradish peroxidase
TBS-T	Tris buffered saline + Triton
TLR	Toll-like receptor
