# Supplementary figures and images for: Additive Behavioral Improvement after Combined Cell Therapy and Rehabilitation Despite Long-Term Microglia Presence in Stroke Rats
Source: Int J Mol Sci. 2021 Feb 3;22(4):1512. doi: 10.3390/ijms22041512 (PMC7913568; doi:10.3390/ijms22041512)

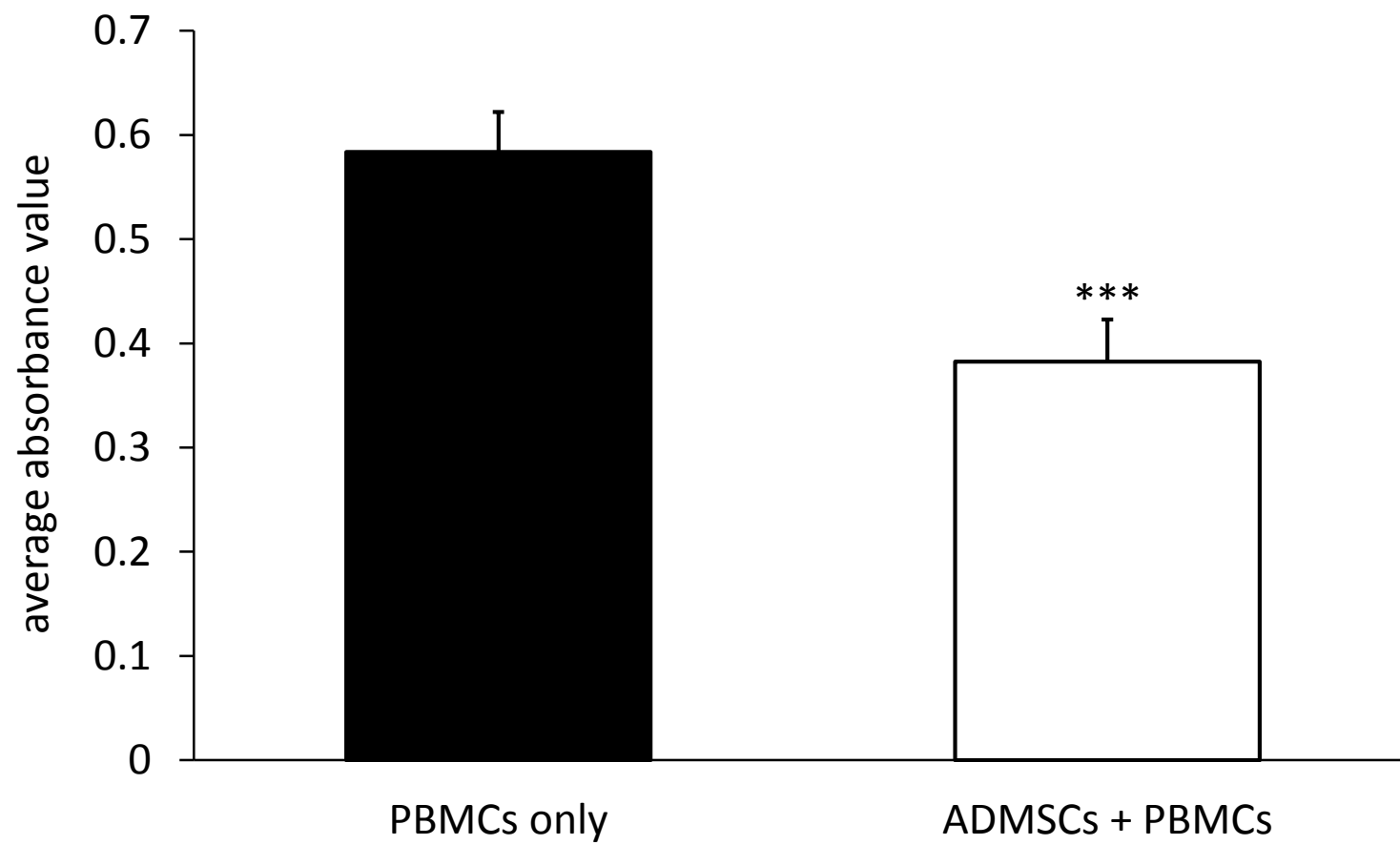

Supplement: Supplementary file 1 [file ijms-22-01512-s001.zip › Figure S1.pdf]

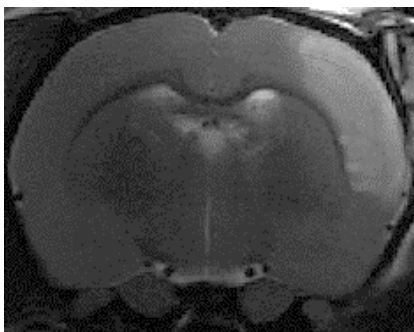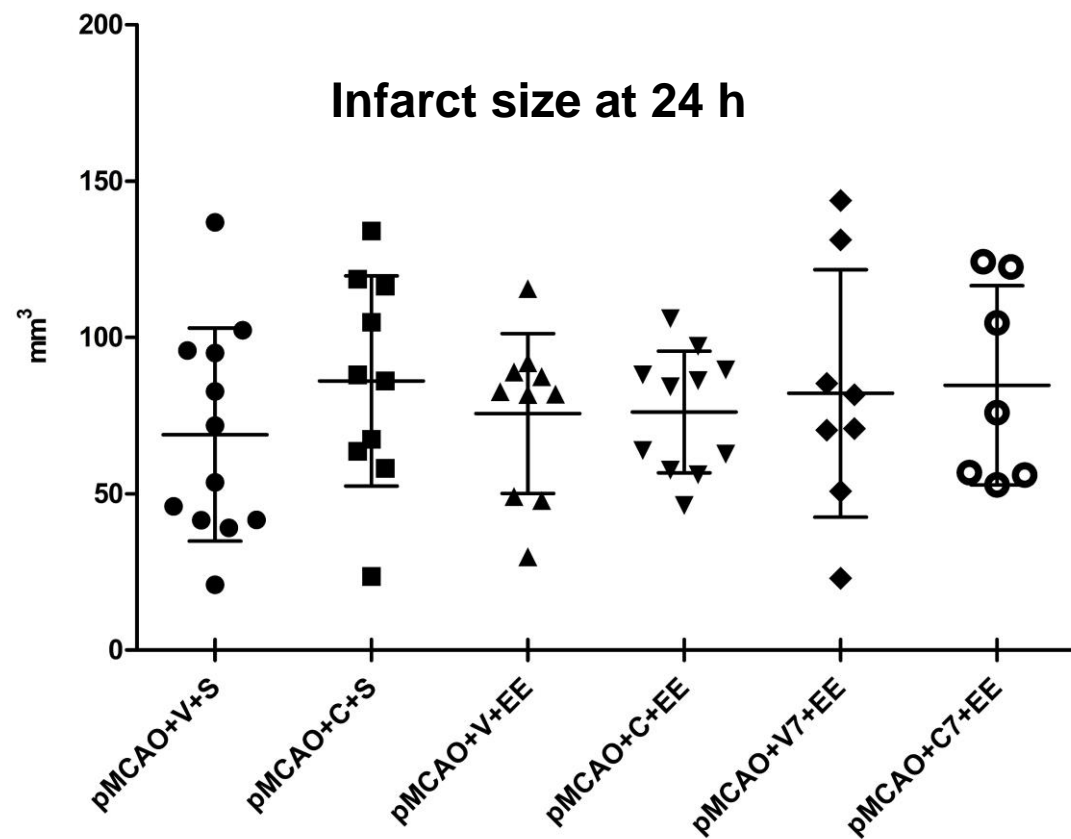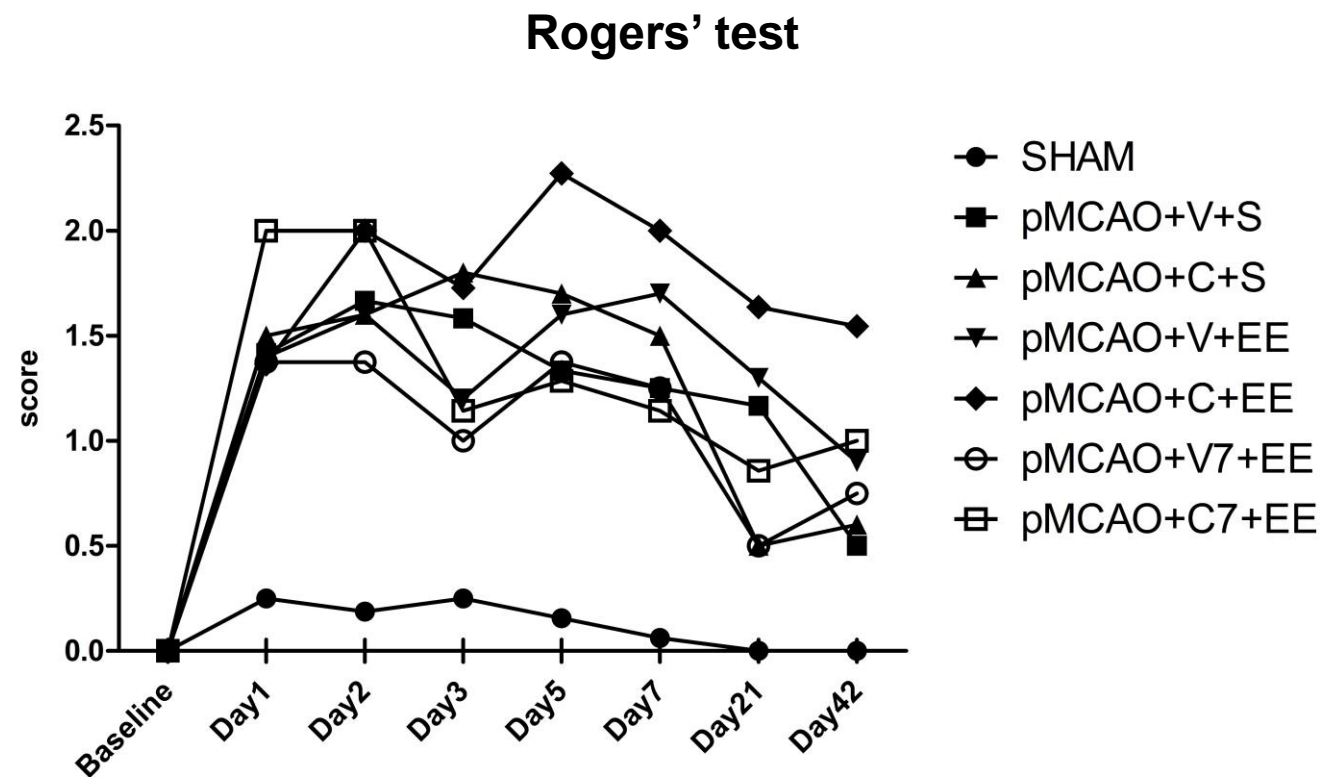

Supplement: Supplementary file 1 [file ijms-22-01512-s001.zip › Figure S2.pdf]
